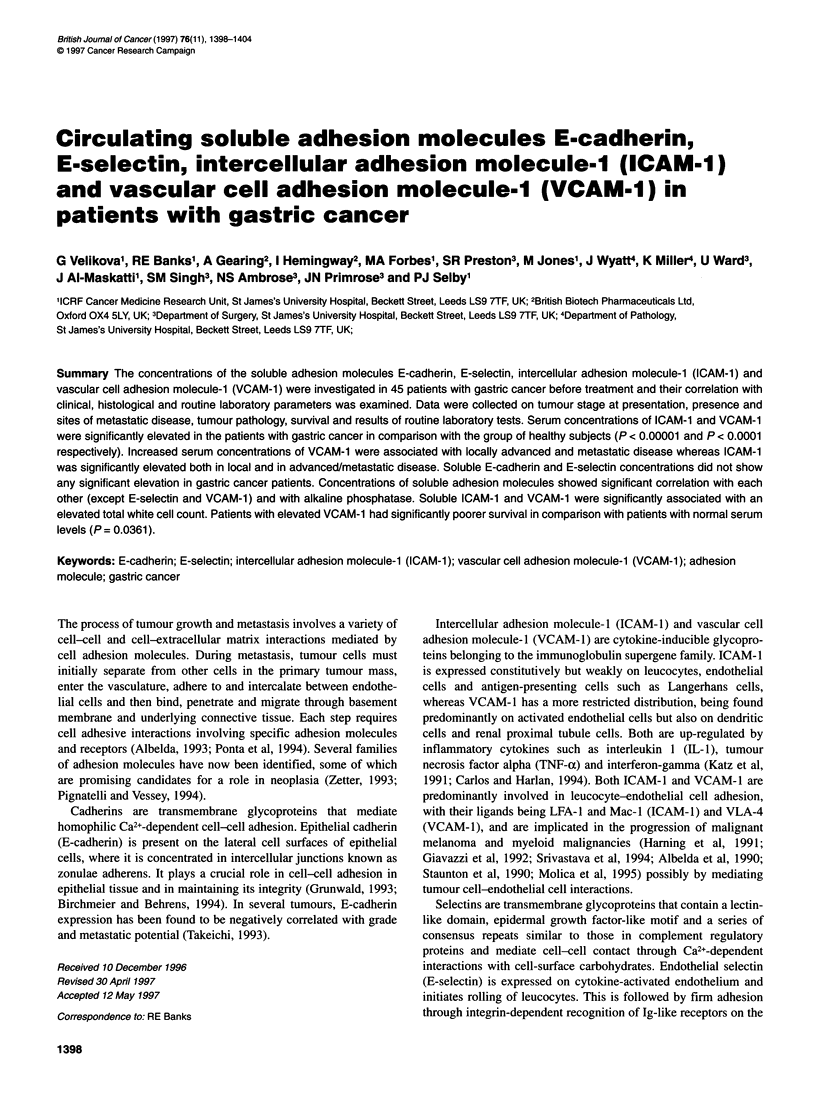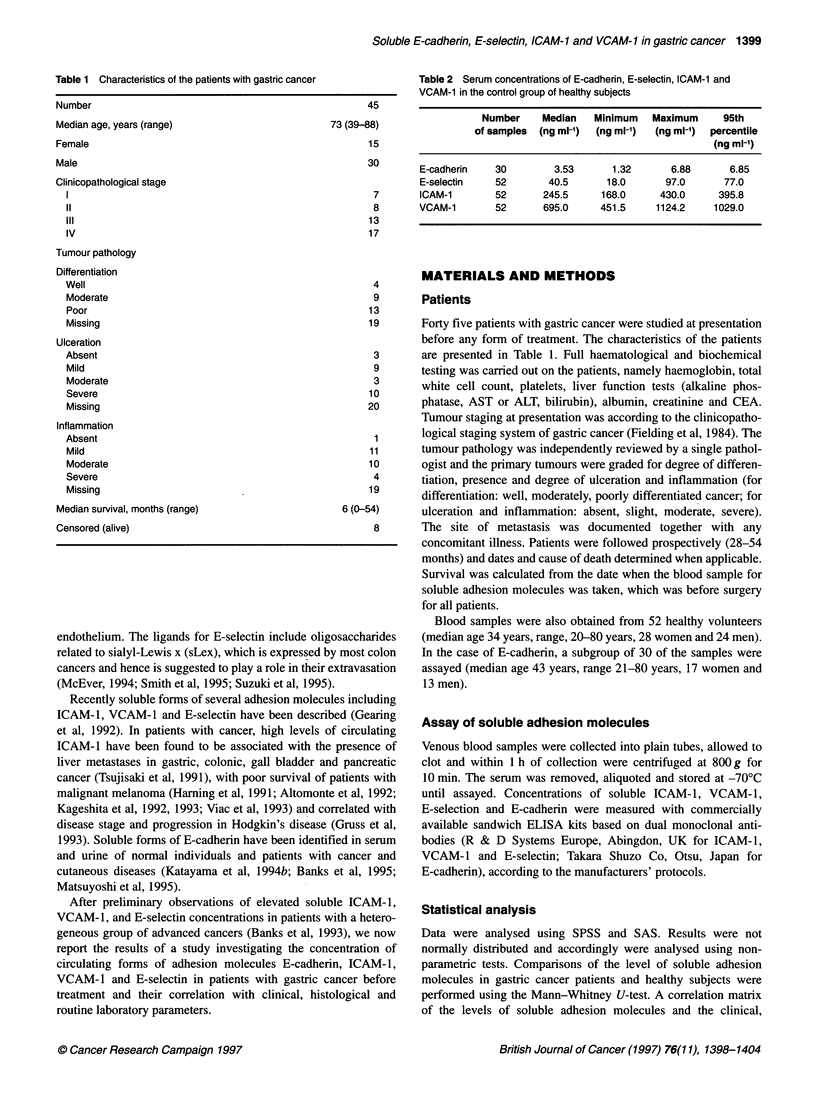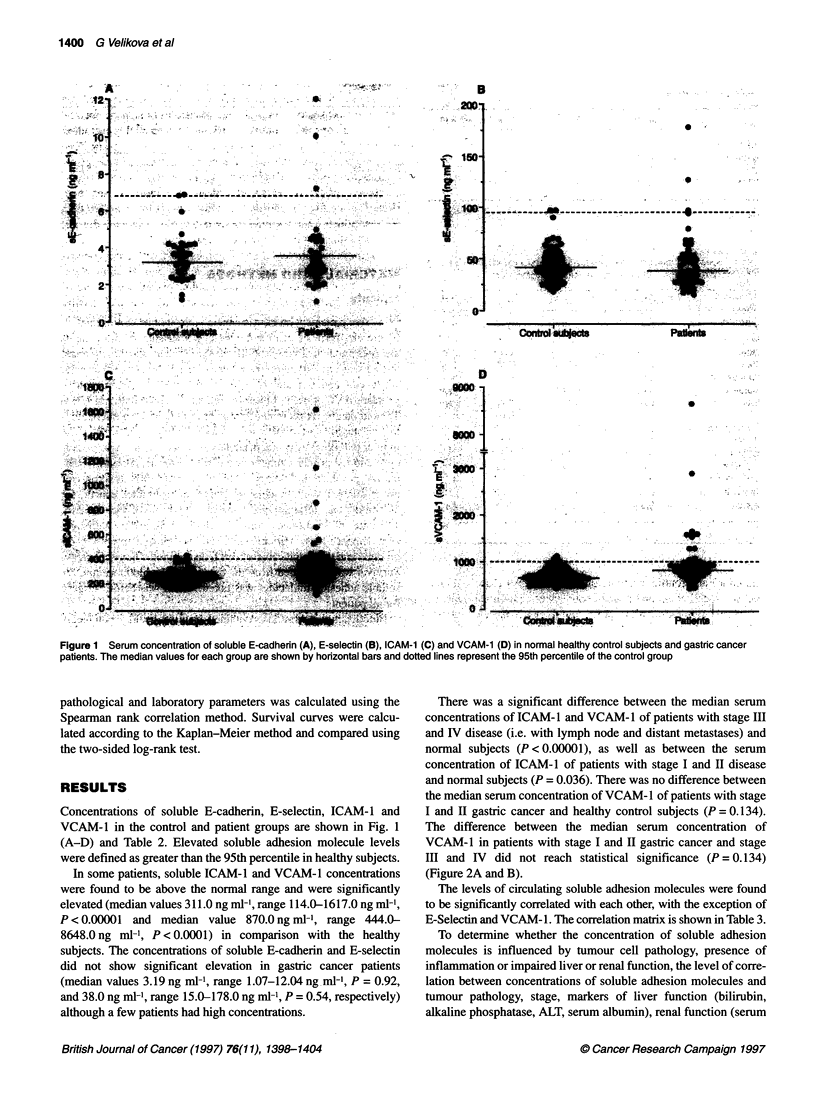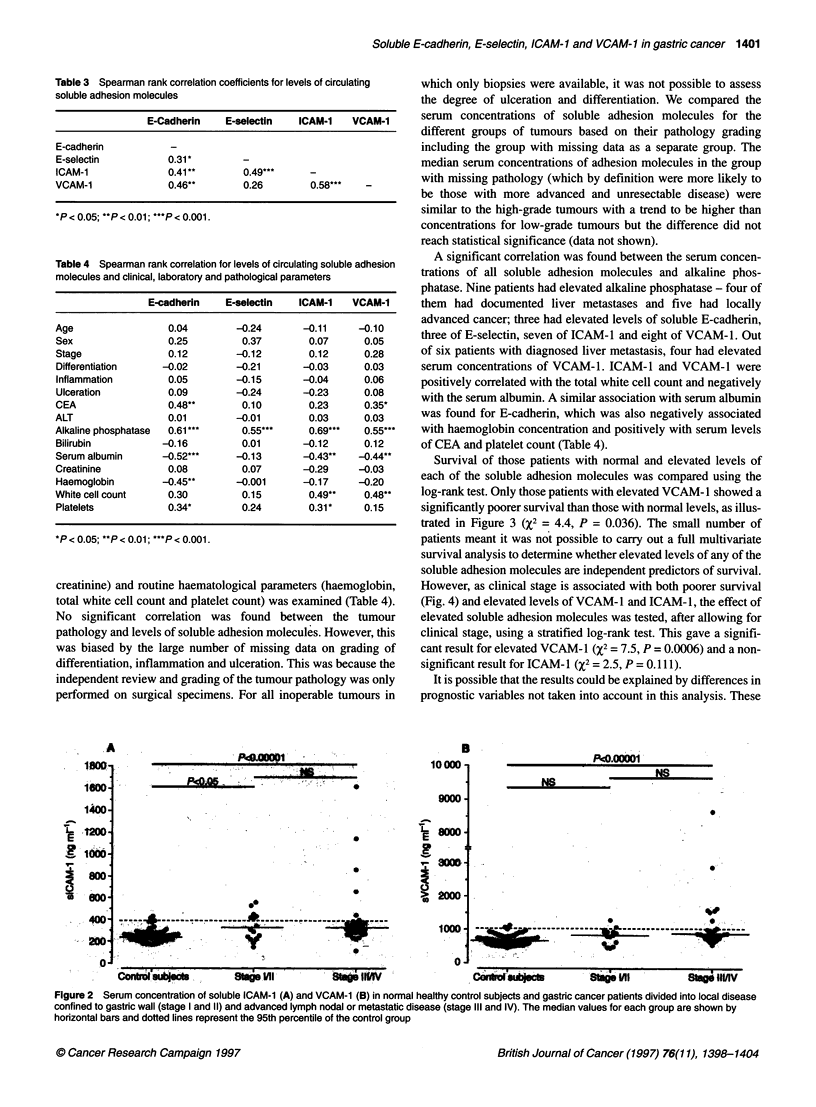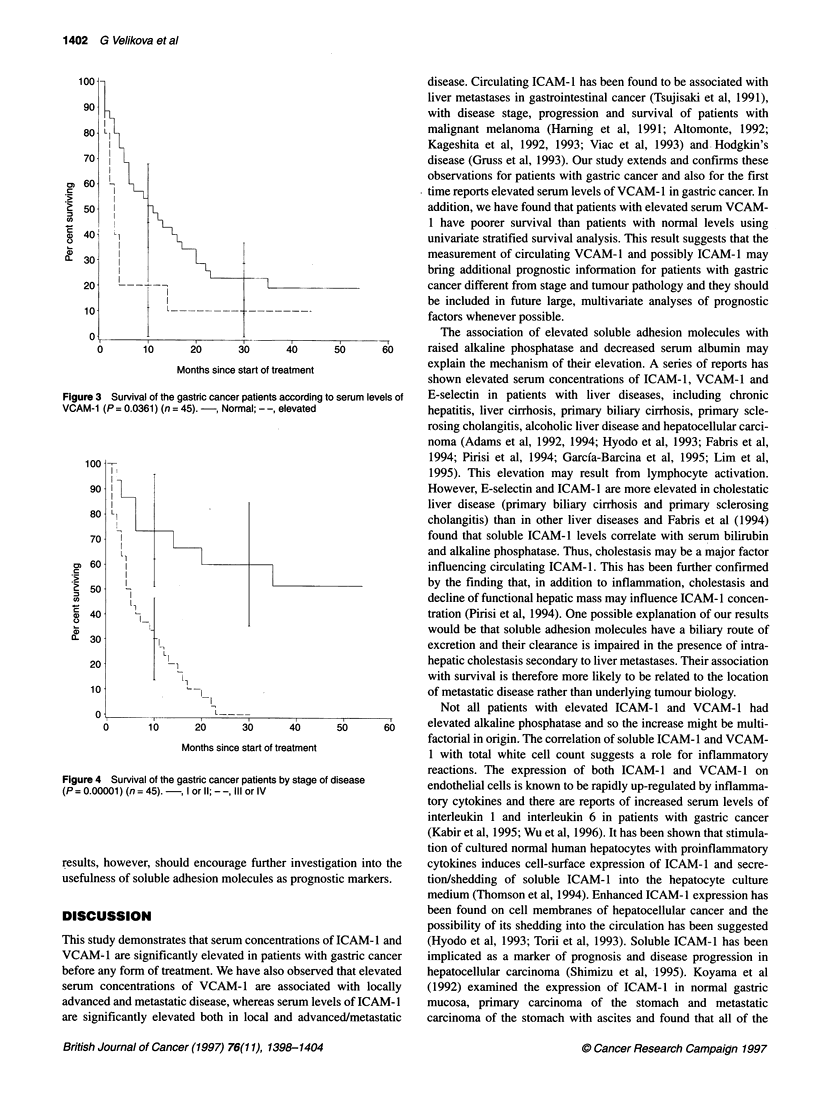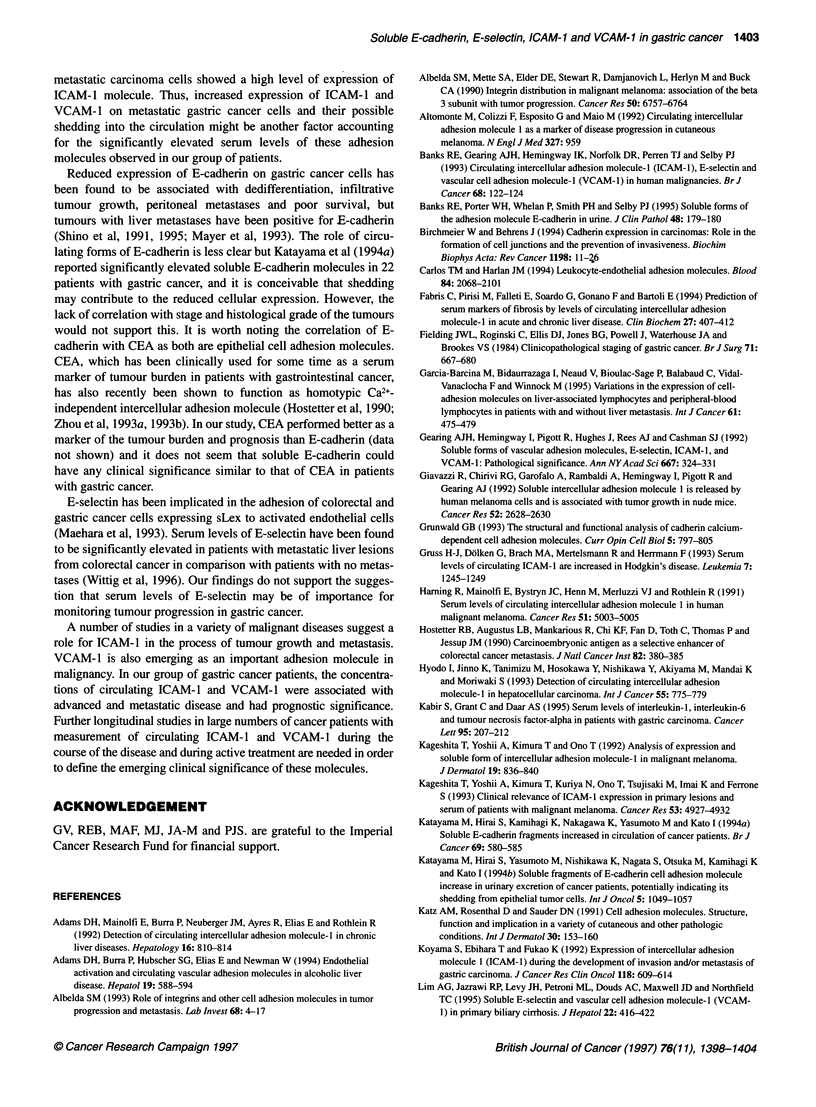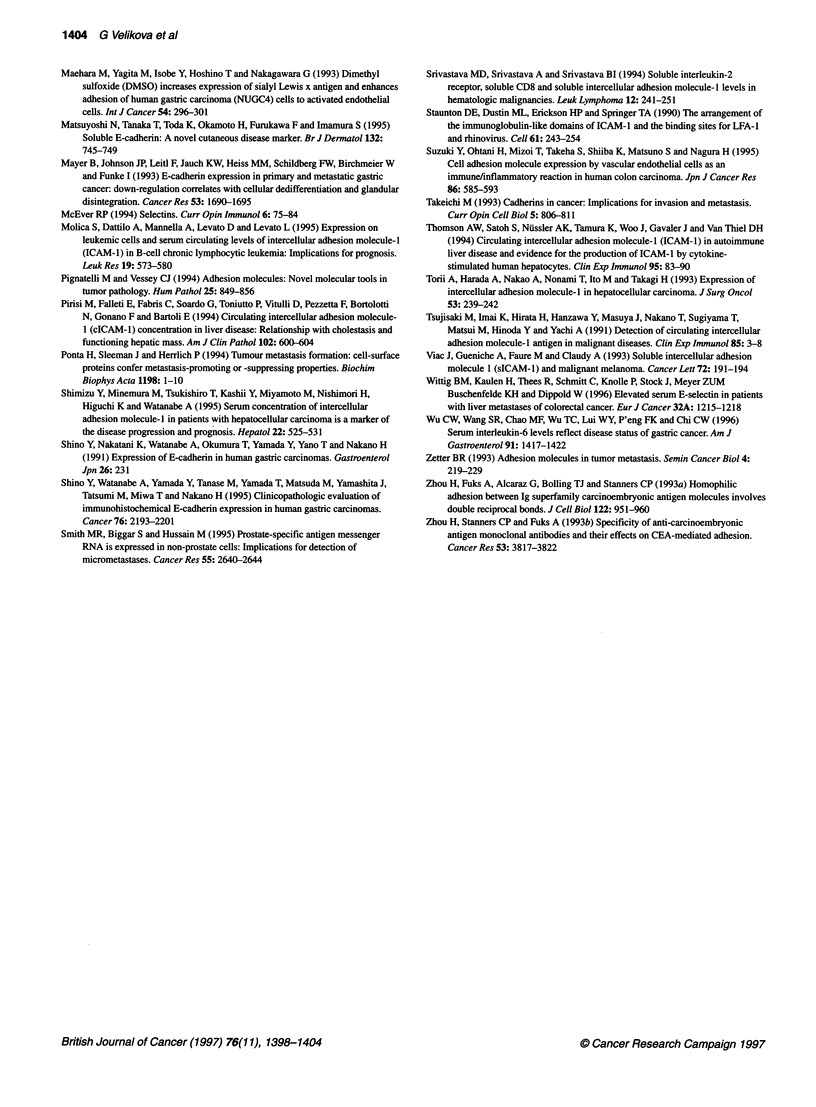# Circulating soluble adhesion molecules E-cadherin, E-selectin, intercellular adhesion molecule-1 (ICAM-1) and vascular cell adhesion molecule-1 (VCAM-1) in patients with gastric cancer.

**DOI:** 10.1038/bjc.1997.569

**Published:** 1997

**Authors:** G. Velikova, R. E. Banks, A. Gearing, I. Hemingway, M. A. Forbes, S. R. Preston, M. Jones, J. Wyatt, K. Miller, U. Ward, J. Al-Maskatti, S. M. Singh, N. S. Ambrose, J. N. Primrose, P. J. Selby

**Affiliations:** ICRF Cancer Medicine Research Unit, St James's University Hospital, Leeds, UK.

## Abstract

**Images:**


British Journal of Cancer (1997) 76(11), 1398–1404
© 1997 Cancer Research Campaign

### Circulating soluble adhesion molecules E-cadherin, E-selectin, intercellular adhesion molecule-1 (ICAM-1) and vascular cell adhesion molecule-1 (VCAM-1) in patients with gastric cancer

G Velikova[1], RE Banks[1], A Gearing[2], I Hemingway[2], MA Forbes[1], SR Preston[3], M Jones[1], J Wyatt[4], K Miller[4], U Ward[3], J Al-Maskatti[1], SM Singh[3], NS Ambrose[3], JN Primrose[3] and PJ Selby[1]

[1]ICRF Cancer Medicine Research Unit, St James's University Hospital, Beckett Street, Leeds LS9 7TF, UK; [2]British Biotech Pharmaceuticals Ltd, Oxford OX4 5LY, UK; [3]Department of Surgery, St James's University Hospital, Beckett Street, Leeds LS9 7TF, UK; [4]Department of Pathology, St James's University Hospital, Beckett Street, Leeds LS9 7TF, UK;

**Summary** The concentrations of the soluble adhesion molecules E-cadherin, E-selectin, intercellular adhesion molecule-1 (ICAM-1) and vascular cell adhesion molecule-1 (VCAM-1) were investigated in 45 patients with gastric cancer before treatment and their correlation with clinical, histological and routine laboratory parameters was examined. Data were collected on tumour stage at presentation, presence and sites of metastatic disease, tumour pathology, survival and results of routine laboratory tests. Serum concentrations of ICAM-1 and VCAM-1 were significantly elevated in the patients with gastric cancer in comparison with the group of healthy subjects ($P < 0.00001$ and $P < 0.0001$ respectively). Increased serum concentrations of VCAM-1 were associated with locally advanced and metastatic disease whereas ICAM-1 was significantly elevated both in local and in advanced/metastatic disease. Soluble E-cadherin and E-selectin concentrations did not show any significant elevation in gastric cancer patients. Concentrations of soluble adhesion molecules showed significant correlation with each other (except E-selectin and VCAM-1) and with alkaline phosphatase. Soluble ICAM-1 and VCAM-1 were significantly associated with an elevated total white cell count. Patients with elevated VCAM-1 had significantly poorer survival in comparison with patients with normal serum levels ($P = 0.0361$).

**Keywords:** E-cadherin; E-selectin; intercellular adhesion molecule-1 (ICAM-1); vascular cell adhesion molecule-1 (VCAM-1); adhesion molecule; gastric cancer

The process of tumour growth and metastasis involves a variety of cell–cell and cell–extracellular matrix interactions mediated by cell adhesion molecules. During metastasis, tumour cells must initially separate from other cells in the primary tumour mass, enter the vasculature, adhere to and intercalate between endothelial cells and then bind, penetrate and migrate through basement membrane and underlying connective tissue. Each step requires cell adhesive interactions involving specific adhesion molecules and receptors (Albelda, 1993; Ponta et al, 1994). Several families of adhesion molecules have now been identified, some of which are promising candidates for a role in neoplasia (Zetter, 1993; Pignatelli and Vessey, 1994).

Cadherins are transmembrane glycoproteins that mediate homophilic $Ca^{2+}$-dependent cell–cell adhesion. Epithelial cadherin (E-cadherin) is present on the lateral cell surfaces of epithelial cells, where it is concentrated in intercellular junctions known as zonulae adherens. It plays a crucial role in cell–cell adhesion in epithelial tissue and in maintaining its integrity (Grunwald, 1993; Birchmeier and Behrens, 1994). In several tumours, E-cadherin expression has been found to be negatively correlated with grade and metastatic potential (Takeichi, 1993).

*Received 10 December 1996*
*Revised 30 April 1997*
*Accepted 12 May 1997*

*Correspondence to:* RE Banks

Intercellular adhesion molecule-1 (ICAM-1) and vascular cell adhesion molecule-1 (VCAM-1) are cytokine-inducible glycoproteins belonging to the immunoglobulin supergene family. ICAM-1 is expressed constitutively but weakly on leucocytes, endothelial cells and antigen-presenting cells such as Langerhans cells, whereas VCAM-1 has a more restricted distribution, being found predominantly on activated endothelial cells but also on dendritic cells and renal proximal tubule cells. Both are up-regulated by inflammatory cytokines such as interleukin 1 (IL-1), tumour necrosis factor alpha (TNF-$\alpha$) and interferon-gamma (Katz et al, 1991; Carlos and Harlan, 1994). Both ICAM-1 and VCAM-1 are predominantly involved in leucocyte–endothelial cell adhesion, with their ligands being LFA-1 and Mac-1 (ICAM-1) and VLA-4 (VCAM-1), and are implicated in the progression of malignant melanoma and myeloid malignancies (Harning et al, 1991; Giavazzi et al, 1992; Srivastava et al, 1994; Albelda et al, 1990; Staunton et al, 1990; Molica et al, 1995) possibly by mediating tumour cell–endothelial cell interactions.

Selectins are transmembrane glycoproteins that contain a lectin-like domain, epidermal growth factor-like motif and a series of consensus repeats similar to those in complement regulatory proteins and mediate cell–cell contact through $Ca^{2+}$-dependent interactions with cell-surface carbohydrates. Endothelial selectin (E-selectin) is expressed on cytokine-activated endothelium and initiates rolling of leucocytes. This is followed by firm adhesion through integrin-dependent recognition of Ig-like receptors on the

**Table 1** Characteristics of the patients with gastric cancer

| | |
|---|---|
| Number | 45 |
| Median age, years (range) | 73 (39–88) |
| Female | 15 |
| Male | 30 |
| Clinicopathological stage | |
| I | 7 |
| II | 8 |
| III | 13 |
| IV | 17 |
| Tumour pathology | |
| Differentiation | |
| Well | 4 |
| Moderate | 9 |
| Poor | 13 |
| Missing | 19 |
| Ulceration | |
| Absent | 3 |
| Mild | 9 |
| Moderate | 3 |
| Severe | 10 |
| Missing | 20 |
| Inflammation | |
| Absent | 1 |
| Mild | 11 |
| Moderate | 10 |
| Severe | 4 |
| Missing | 19 |
| Median survival, months (range) | 6 (0–54) |
| Censored (alive) | 8 |

**Table 2** Serum concentrations of E-cadherin, E-selectin, ICAM-1 and VCAM-1 in the control group of healthy subjects

| | Number of samples | Median (ng ml$^{-1}$) | Minimum (ng ml$^{-1}$) | Maximum (ng ml$^{-1}$) | 95th percentile (ng ml$^{-1}$) |
|---|---|---|---|---|---|
| E-cadherin | 30 | 3.53 | 1.32 | 6.88 | 6.85 |
| E-selectin | 52 | 40.5 | 18.0 | 97.0 | 77.0 |
| ICAM-1 | 52 | 245.5 | 168.0 | 430.0 | 395.8 |
| VCAM-1 | 52 | 695.0 | 451.5 | 1124.2 | 1029.0 |

endothelium. The ligands for E-selectin include oligosaccharides related to sialyl-Lewis x (sLex), which is expressed by most colon cancers and hence is suggested to play a role in their extravasation (McEver, 1994; Smith et al, 1995; Suzuki et al, 1995).

Recently soluble forms of several adhesion molecules including ICAM-1, VCAM-1 and E-selectin have been described (Gearing et al, 1992). In patients with cancer, high levels of circulating ICAM-1 have been found to be associated with the presence of liver metastases in gastric, colonic, gall bladder and pancreatic cancer (Tsujisaki et al, 1991), with poor survival of patients with malignant melanoma (Harning et al, 1991; Altomonte et al, 1992; Kageshita et al, 1992, 1993; Viac et al, 1993) and correlated with disease stage and progression in Hodgkin's disease (Gruss et al, 1993). Soluble forms of E-cadherin have been identified in serum and urine of normal individuals and patients with cancer and cutaneous diseases (Katayama et al, 1994*b*; Banks et al, 1995; Matsuyoshi et al, 1995).

After preliminary observations of elevated soluble ICAM-1, VCAM-1, and E-selectin concentrations in patients with a heterogeneous group of advanced cancers (Banks et al, 1993), we now report the results of a study investigating the concentration of circulating forms of adhesion molecules E-cadherin, ICAM-1, VCAM-1 and E-selectin in patients with gastric cancer before treatment and their correlation with clinical, histological and routine laboratory parameters.

### MATERIALS AND METHODS

### Patients

Forty five patients with gastric cancer were studied at presentation before any form of treatment. The characteristics of the patients are presented in Table 1. Full haematological and biochemical testing was carried out on the patients, namely haemoglobin, total white cell count, platelets, liver function tests (alkaline phosphatase, AST or ALT, bilirubin), albumin, creatinine and CEA. Tumour staging at presentation was according to the clinicopathological staging system of gastric cancer (Fielding et al, 1984). The tumour pathology was independently reviewed by a single pathologist and the primary tumours were graded for degree of differentiation, presence and degree of ulceration and inflammation (for differentiation: well, moderately, poorly differentiated cancer; for ulceration and inflammation: absent, slight, moderate, severe). The site of metastasis was documented together with any concomitant illness. Patients were followed prospectively (28–54 months) and dates and cause of death determined when applicable. Survival was calculated from the date when the blood sample for soluble adhesion molecules was taken, which was before surgery for all patients.

Blood samples were also obtained from 52 healthy volunteers (median age 34 years, range, 20–80 years, 28 women and 24 men). In the case of E-cadherin, a subgroup of 30 of the samples were assayed (median age 43 years, range 21–80 years, 17 women and 13 men).

### Assay of soluble adhesion molecules

Venous blood samples were collected into plain tubes, allowed to clot and within 1 h of collection were centrifuged at 800 *g* for 10 min. The serum was removed, aliquoted and stored at –70°C until assayed. Concentrations of soluble ICAM-1, VCAM-1, E-selection and E-cadherin were measured with commercially available sandwich ELISA kits based on dual monoclonal antibodies (R & D Systems Europe, Abingdon, UK for ICAM-1, VCAM-1 and E-selectin; Takara Shuzo Co, Otsu, Japan for E-cadherin), according to the manufacturers' protocols.

### Statistical analysis

Data were analysed using SPSS and SAS. Results were not normally distributed and accordingly were analysed using non-parametric tests. Comparisons of the level of soluble adhesion molecules in gastric cancer patients and healthy subjects were performed using the Mann–Whitney *U*-test. A correlation matrix of the levels of soluble adhesion molecules and the clinical,

© Cancer Research Campaign 1997 

**Figure 1** Serum concentration of soluble E-cadherin (**A**), E-selectin (**B**), ICAM-1 (**C**) and VCAM-1 (**D**) in normal healthy control subjects and gastric cancer patients. The median values for each group are shown by horizontal bars and dotted lines represent the 95th percentile of the control group pathological and laboratory parameters was calculated using the Spearman rank correlation method. Survival curves were calculated according to the Kaplan–Meier method and compared using the two-sided log-rank test.

### RESULTS

Concentrations of soluble E-cadherin, E-selectin, ICAM-1 and VCAM-1 in the control and patient groups are shown in Fig. 1 (A–D) and Table 2. Elevated soluble adhesion molecule levels were defined as greater than the 95th percentile in healthy subjects.

In some patients, soluble ICAM-1 and VCAM-1 concentrations were found to be above the normal range and were significantly elevated (median values 311.0 ng $ml^{-1}$, range 114.0–1617.0 ng $ml^{-1}$, $P < 0.00001$ and median value 870.0 ng $ml^{-1}$, range 444.0–8648.0 ng $ml^{-1}$, $P < 0.0001$) in comparison with the healthy subjects. The concentrations of soluble E-cadherin and E-selectin did not show significant elevation in gastric cancer patients (median values 3.19 ng $ml^{-1}$, range 1.07–12.04 ng $ml^{-1}$, $P = 0.92$, and 38.0 ng $ml^{-1}$, range 15.0–178.0 ng $ml^{-1}$, $P = 0.54$, respectively) although a few patients had high concentrations.

There was a significant difference between the median serum concentrations of ICAM-1 and VCAM-1 of patients with stage III and IV disease (i.e. with lymph node and distant metastases) and normal subjects ($P < 0.00001$), as well as between the serum concentration of ICAM-1 of patients with stage I and II disease and normal subjects ($P = 0.036$). There was no difference between the median serum concentration of VCAM-1 of patients with stage I and II gastric cancer and healthy control subjects ($P = 0.134$). The difference between the median serum concentration of VCAM-1 in patients with stage I and II gastric cancer and stage III and IV did not reach statistical significance ($P = 0.134$) (Figure 2A and B).

The levels of circulating soluble adhesion molecules were found to be significantly correlated with each other, with the exception of E-Selectin and VCAM-1. The correlation matrix is shown in Table 3.

To determine whether the concentration of soluble adhesion molecules is influenced by tumour cell pathology, presence of inflammation or impaired liver or renal function, the level of correlation between concentrations of soluble adhesion molecules and tumour pathology, stage, markers of liver function (bilirubin, alkaline phosphatase, ALT, serum albumin), renal function (serum

© Cancer Research Campaign 1997

**Table 3** Spearman rank correlation coefficients for levels of circulating soluble adhesion molecules

| | E-Cadherin | E-selectin | ICAM-1 | VCAM-1 |
|---|---|---|---|---|
| E-cadherin | – | | | |
| E-selectin | 0.31* | – | | |
| ICAM-1 | 0.41** | 0.49*** | – | |
| VCAM-1 | 0.46** | 0.26 | 0.58*** | – |

*$P < 0.05$; **$P < 0.01$; ***$P < 0.001$.

**Table 4** Spearman rank correlation for levels of circulating soluble adhesion molecules and clinical, laboratory and pathological parameters

| | E-cadherin | E-selectin | ICAM-1 | VCAM-1 |
|---|---|---|---|---|
| Age | 0.04 | –0.24 | –0.11 | –0.10 |
| Sex | 0.25 | 0.37 | 0.07 | 0.05 |
| Stage | 0.12 | –0.12 | 0.12 | 0.28 |
| Differentiation | –0.02 | –0.21 | –0.03 | 0.03 |
| Inflammation | 0.05 | –0.15 | –0.04 | 0.06 |
| Ulceration | 0.09 | –0.24 | –0.23 | 0.08 |
| CEA | 0.48** | 0.10 | 0.23 | 0.35* |
| ALT | 0.01 | –0.01 | 0.03 | 0.03 |
| Alkaline phosphatase | 0.61*** | 0.55*** | 0.69*** | 0.55*** |
| Bilirubin | –0.16 | 0.01 | –0.12 | 0.12 |
| Serum albumin | –0.52*** | –0.13 | –0.43** | –0.44** |
| Creatinine | 0.08 | 0.07 | –0.29 | –0.03 |
| Haemoglobin | –0.45** | –0.001 | –0.17 | –0.20 |
| White cell count | 0.30 | 0.15 | 0.49** | 0.48** |
| Platelets | 0.34* | 0.24 | 0.31* | 0.15 |

*$P < 0.05$; **$P < 0.01$; ***$P < 0.001$.

creatinine) and routine haematological parameters (haemoglobin, total white cell count and platelet count) was examined (Table 4). No significant correlation was found between the tumour pathology and levels of soluble adhesion molecules. However, this was biased by the large number of missing data on grading of differentiation, inflammation and ulceration. This was because the independent review and grading of the tumour pathology was only performed on surgical specimens. For all inoperable tumours in which only biopsies were available, it was not possible to assess the degree of ulceration and differentiation. We compared the serum concentrations of soluble adhesion molecules for the different groups of tumours based on their pathology grading including the group with missing data as a separate group. The median serum concentrations of adhesion molecules in the group with missing pathology (which by definition were more likely to be those with more advanced and unresectable disease) were similar to the high-grade tumours with a trend to be higher than concentrations for low-grade tumours but the difference did not reach statistical significance (data not shown).

A significant correlation was found between the serum concentrations of all soluble adhesion molecules and alkaline phosphatase. Nine patients had elevated alkaline phosphatase – four of them had documented liver metastases and five had locally advanced cancer; three had elevated levels of soluble E-cadherin, three of E-selectin, seven of ICAM-1 and eight of VCAM-1. Out of six patients with diagnosed liver metastasis, four had elevated serum concentrations of VCAM-1. ICAM-1 and VCAM-1 were positively correlated with the total white cell count and negatively with the serum albumin. A similar association with serum albumin was found for E-cadherin, which was also negatively associated with haemoglobin concentration and positively with serum levels of CEA and platelet count (Table 4).

Survival of those patients with normal and elevated levels of each of the soluble adhesion molecules was compared using the log-rank test. Only those patients with elevated VCAM-1 showed a significantly poorer survival than those with normal levels, as illustrated in Figure 3 ($\chi^2 = 4.4$, $P = 0.036$). The small number of patients meant it was not possible to carry out a full multivariate survival analysis to determine whether elevated levels of any of the soluble adhesion molecules are independent predictors of survival. However, as clinical stage is associated with both poorer survival (Fig. 4) and elevated levels of VCAM-1 and ICAM-1, the effect of elevated soluble adhesion molecules was tested, after allowing for clinical stage, using a stratified log-rank test. This gave a significant result for elevated VCAM-1 ($\chi^2 = 7.5$, $P = 0.0006$) and a non-significant result for ICAM-1 ($\chi^2 = 2.5$, $P = 0.111$).

It is possible that the results could be explained by differences in prognostic variables not taken into account in this analysis. These

**Figure 2** Serum concentration of soluble ICAM-1 (**A**) and VCAM-1 (**B**) in normal healthy control subjects and gastric cancer patients divided into local disease confined to gastric wall (stage I and II) and advanced lymph nodal or metastatic disease (stage III and IV). The median values for each group are shown by horizontal bars and dotted lines represent the 95th percentile of the control group

**Figure 3** Survival of the gastric cancer patients according to serum levels of VCAM-1 ($P$ = 0.0361) ($n$ = 45). ——, Normal; – –, elevated

**Figure 4** Survival of the gastric cancer patients by stage of disease ($P$ = 0.00001) ($n$ = 45). ——, I or II; – –, III or IV

results, however, should encourage further investigation into the usefulness of soluble adhesion molecules as prognostic markers.

### DISCUSSION

This study demonstrates that serum concentrations of ICAM-1 and VCAM-1 are significantly elevated in patients with gastric cancer before any form of treatment. We have also observed that elevated serum concentrations of VCAM-1 are associated with locally advanced and metastatic disease, whereas serum levels of ICAM-1 are significantly elevated both in local and advanced/metastatic disease. Circulating ICAM-1 has been found to be associated with liver metastases in gastrointestinal cancer (Tsujisaki et al, 1991), with disease stage, progression and survival of patients with malignant melanoma (Harning et al, 1991; Altomonte, 1992; Kageshita et al, 1992, 1993; Viac et al, 1993) and Hodgkin's disease (Gruss et al, 1993). Our study extends and confirms these observations for patients with gastric cancer and also for the first time reports elevated serum levels of VCAM-1 in gastric cancer. In addition, we have found that patients with elevated serum VCAM-1 have poorer survival than patients with normal levels using univariate stratified survival analysis. This result suggests that the measurement of circulating VCAM-1 and possibly ICAM-1 may bring additional prognostic information for patients with gastric cancer different from stage and tumour pathology and they should be included in future large, multivariate analyses of prognostic factors whenever possible.

The association of elevated soluble adhesion molecules with raised alkaline phosphatase and decreased serum albumin may explain the mechanism of their elevation. A series of reports has shown elevated serum concentrations of ICAM-1, VCAM-1 and E-selectin in patients with liver diseases, including chronic hepatitis, liver cirrhosis, primary biliary cirrhosis, primary sclerosing cholangitis, alcoholic liver disease and hepatocellular carcinoma (Adams et al, 1992, 1994; Hyodo et al, 1993; Fabris et al, 1994; Pirisi et al, 1994; García-Barcina et al, 1995; Lim et al, 1995). This elevation may result from lymphocyte activation. However, E-selectin and ICAM-1 are more elevated in cholestatic liver disease (primary biliary cirrhosis and primary sclerosing cholangitis) than in other liver diseases and Fabris et al (1994) found that soluble ICAM-1 levels correlate with serum bilirubin and alkaline phosphatase. Thus, cholestasis may be a major factor influencing circulating ICAM-1. This has been further confirmed by the finding that, in addition to inflammation, cholestasis and decline of functional hepatic mass may influence ICAM-1 concentration (Pirisi et al, 1994). One possible explanation of our results would be that soluble adhesion molecules have a biliary route of excretion and their clearance is impaired in the presence of intrahepatic cholestasis secondary to liver metastases. Their association with survival is therefore more likely to be related to the location of metastatic disease rather than underlying tumour biology.

Not all patients with elevated ICAM-1 and VCAM-1 had elevated alkaline phosphatase and so the increase might be multifactorial in origin. The correlation of soluble ICAM-1 and VCAM-1 with total white cell count suggests a role for inflammatory reactions. The expression of both ICAM-1 and VCAM-1 on endothelial cells is known to be rapidly up-regulated by inflammatory cytokines and there are reports of increased serum levels of interleukin 1 and interleukin 6 in patients with gastric cancer (Kabir et al, 1995; Wu et al, 1996). It has been shown that stimulation of cultured normal human hepatocytes with proinflammatory cytokines induces cell-surface expression of ICAM-1 and secretion/shedding of soluble ICAM-1 into the hepatocyte culture medium (Thomson et al, 1994). Enhanced ICAM-1 expression has been found on cell membranes of hepatocellular cancer and the possibility of its shedding into the circulation has been suggested (Hyodo et al, 1993; Torii et al, 1993). Soluble ICAM-1 has been implicated as a marker of prognosis and disease progression in hepatocellular carcinoma (Shimizu et al, 1995). Koyama et al (1992) examined the expression of ICAM-1 in normal gastric mucosa, primary carcinoma of the stomach and metastatic carcinoma of the stomach with ascites and found that all of the

© Cancer Research Campaign 1997

metastatic carcinoma cells showed a high level of expression of ICAM-1 molecule. Thus, increased expression of ICAM-1 and VCAM-1 on metastatic gastric cancer cells and their possible shedding into the circulation might be another factor accounting for the significantly elevated serum levels of these adhesion molecules observed in our group of patients.

Reduced expression of E-cadherin on gastric cancer cells has been found to be associated with dedifferentiation, infiltrative tumour growth, peritoneal metastases and poor survival, but tumours with liver metastases have been positive for E-cadherin (Shino et al, 1991, 1995; Mayer et al, 1993). The role of circulating forms of E-cadherin is less clear but Katayama et al (1994*a*) reported significantly elevated soluble E-cadherin molecules in 22 patients with gastric cancer, and it is conceivable that shedding may contribute to the reduced cellular expression. However, the lack of correlation with stage and histological grade of the tumours would not support this. It is worth noting the correlation of E-cadherin with CEA as both are epithelial cell adhesion molecules. CEA, which has been clinically used for some time as a serum marker of tumour burden in patients with gastrointestinal cancer, has also recently been shown to function as homotypic $Ca^{2+}$-independent intercellular adhesion molecule (Hostetter et al, 1990; Zhou et al, 1993*a*, 1993b). In our study, CEA performed better as a marker of the tumour burden and prognosis than E-cadherin (data not shown) and it does not seem that soluble E-cadherin could have any clinical significance similar to that of CEA in patients with gastric cancer.

E-selectin has been implicated in the adhesion of colorectal and gastric cancer cells expressing sLex to activated endothelial cells (Maehara et al, 1993). Serum levels of E-selectin have been found to be significantly elevated in patients with metastatic liver lesions from colorectal cancer in comparison with patients with no metastases (Wittig et al, 1996). Our findings do not support the suggestion that serum levels of E-selectin may be of importance for monitoring tumour progression in gastric cancer.

A number of studies in a variety of malignant diseases suggest a role for ICAM-1 in the process of tumour growth and metastasis. VCAM-1 is also emerging as an important adhesion molecule in malignancy. In our group of gastric cancer patients, the concentrations of circulating ICAM-1 and VCAM-1 were associated with advanced and metastatic disease and had prognostic significance. Further longitudinal studies in large numbers of cancer patients with measurement of circulating ICAM-1 and VCAM-1 during the course of the disease and during active treatment are needed in order to define the emerging clinical significance of these molecules.

### ACKNOWLEDGEMENT

GV, REB, MAF, MJ, JA-M and PJS. are grateful to the Imperial Cancer Research Fund for financial support.

### REFERENCES

Adams DH, Mainolfi E, Burra P, Neuberger JM, Ayres R, Elias E and Rothlein R (1992) Detection of circulating intercellular adhesion molecule-1 in chronic liver diseases. *Hepatology* **16:** 810–814

Adams DH, Burra P, Hubscher SG, Elias E and Newman W (1994) Endothelial activation and circulating vascular adhesion molecules in alcoholic liver disease. *Hepatol* **19:** 588–594

Albelda SM (1993) Role of integrins and other cell adhesion molecules in tumor progression and metastasis. *Lab Invest* **68:** 4–17

Albelda SM, Mette SA, Elder DE, Stewart R, Damjanovich L, Herlyn M and Buck CA (1990) Integrin distribution in malignant melanoma: association of the beta 3 subunit with tumor progression. *Cancer Res* **50:** 6757–6764

Altomonte M, Colizzi F, Esposito G and Maio M (1992) Circulating intercellular adhesion molecule 1 as a marker of disease progression in cutaneous melanoma. *N Engl J Med* **327:** 959

Banks RE, Gearing AJH, Hemingway IK, Norfolk DR, Perren TJ and Selby PJ (1993) Circulating intercellular adhesion molecule-1 (ICAM-1), E-selectin and vascular cell adhesion molecule-1 (VCAM-1) in human malignancies. *Br J Cancer* **68:** 122–124

Banks RE, Porter WH, Whelan P, Smith PH and Selby PJ (1995) Soluble forms of the adhesion molecule E-cadherin in urine. *J Clin Pathol* **48:** 179–180

Birchmeier W and Behrens J (1994) Cadherin expression in carcinomas: Role in the formation of cell junctions and the prevention of invasiveness. *Biochim Biophys Acta: Rev Cancer* **1198:** 11–26

Carlos TM and Harlan JM (1994) Leukocyte-endothelial adhesion molecules. *Blood* **84:** 2068–2101

Fabris C, Pirisi M, Falleti E, Soardo G, Gonano F and Bartoli E (1994) Prediction of serum markers of fibrosis by levels of circulating intercellular adhesion molecule-1 in acute and chronic liver disease. *Clin Biochem* **27:** 407–412

Fielding JWL, Roginski C, Ellis DJ, Jones BG, Powell J, Waterhouse JA and Brookes VS (1984) Clinicopathological staging of gastric cancer. *Br J Surg* **71:** 667–680

Garcia-Barcina M, Bidaurrazaga I, Neaud V, Bioulac-Sage P, Balabaud C, Vidal-Vanaclocha F and Winnock M (1995) Variations in the expression of cell-adhesion molecules on liver-associated lymphocytes and peripheral-blood lymphocytes in patients with and without liver metastasis. *Int J Cancer* **61:** 475–479

Gearing AJH, Hemingway I, Pigott R, Hughes J, Rees AJ and Cashman SJ (1992) Soluble forms of vascular adhesion molecules, E-selectin, ICAM-1, and VCAM-1: Pathological significance. *Ann NY Acad Sci* **667:** 324–331

Giavazzi R, Chirivi RG, Garofalo A, Rambaldi A, Hemingway I, Pigott R and Gearing AJ (1992) Soluble intercellular adhesion molecule 1 is released by human melanoma cells and is associated with tumor growth in nude mice. *Cancer Res* **52:** 2628–2630

Grunwald GB (1993) The structural and functional analysis of cadherin calcium-dependent cell adhesion molecules. *Curr Opin Cell Biol* **5:** 797–805

Gruss H-J, Dölken G, Brach MA, Mertelsmann R and Herrmann F (1993) Serum levels of circulating ICAM-1 are increased in Hodgkin's disease. *Leukemia* **7:** 1245–1249

Harning R, Mainolfi E, Bystryn JC, Henn M, Merluzzi VJ and Rothlein R (1991) Serum levels of circulating intercellular adhesion molecule 1 in human malignant melanoma. *Cancer Res* **51:** 5003–5005

Hostetter RB, Augustus LB, Mankarious R, Chi KF, Fan D, Toth C, Thomas P and Jessup JM (1990) Carcinoembryonic antigen as a selective enhancer of colorectal cancer metastasis. *J Natl Cancer Inst* **82:** 380–385

Hyodo I, Jinno K, Tanimizu M, Hosokawa Y, Nishikawa Y, Akiyama M, Mandai K and Moriwaki S (1993) Detection of circulating intercellular adhesion molecule-1 in hepatocellular carcinoma. *Int J Cancer* **55:** 775–779

Kabir S, Grant C and Daar AS (1995) Serum levels of interleukin-1, interleukin-6 and tumour necrosis factor-alpha in patients with gastric carcinoma. *Cancer Lett* **95:** 207–212

Kageshita T, Yoshii A, Kimura T and Ono T (1992) Analysis of expression and soluble form of intercellular adhesion molecule-1 in malignant melanoma. *J Dermatol* **19:** 836–840

Kageshita T, Yoshii A, Kimura T, Kuriya N, Ono T, Tsujisaki M, Imai K and Ferrone S (1993) Clinical relevance of ICAM-1 expression in primary lesions and serum of patients with malignant melanoma. *Cancer Res* **53:** 4927–4932

Katayama M, Hirai S, Kamihagi K, Nakagawa K, Yasumoto M and Kato I (1994*a*) Soluble E-cadherin fragments increased in circulation of cancer patients. *Br J Cancer* **69:** 580–585

Katayama M, Hirai S, Yasumoto M, Nishikawa K, Nagata S, Otsuka M, Kamihagi K and Kato I (1994*b*) Soluble fragments of E-cadherin cell adhesion molecule increase in urinary excretion of cancer patients, potentially indicating its shedding from epithelial tumor cells. *Int J Oncol* **5:** 1049–1057

Katz AM, Rosenthal D and Sauder DN (1991) Cell adhesion molecules. Structure, function and implication in a variety of cutaneous and other pathologic conditions. *Int J Dermatol* **30:** 153–160

Koyama S, Ebihara T and Fukao K (1992) Expression of intercellular adhesion molecule 1 (ICAM-1) during the development of invasion and/or metastasis of gastric carcinoma. *J Cancer Res Clin Oncol* **118:** 609–614

Lim AG, Jazrawi RP, Levy JH, Petroni ML, Douds AC, Maxwell JD and Northfield TC (1995) Soluble E-selectin and vascular cell adhesion molecule-1 (VCAM-1) in primary biliary cirrhosis. *J Hepatol* **22:** 416–422

© Cancer Research Campaign 1997 

Maehara M, Yagita M, Isobe Y, Hoshino T and Nakagawara G (1993) Dimethyl sulfoxide (DMSO) increases expression of sialyl Lewis x antigen and enhances adhesion of human gastric carcinoma (NUGC4) cells to activated endothelial cells. *Int J Cancer* **54**: 296–301

Matsuyoshi N, Tanaka T, Toda K, Okamoto H, Furukawa F and Imamura S (1995) Soluble E-cadherin: A novel cutaneous disease marker. *Br J Dermatol* **132**: 745–749

Mayer B, Johnson JP, Leitl F, Jauch KW, Heiss MM, Schildberg FW, Birchmeier W and Funke I (1993) E-cadherin expression in primary and metastatic gastric cancer: down-regulation correlates with cellular dedifferentiation and glandular disintegration. *Cancer Res* **53**: 1690–1695

McEver RP (1994) Selectins. *Curr Opin Immunol* **6**: 75–84

Molica S, Dattilo A, Mannella A, Levato D and Levato L (1995) Expression on leukemic cells and serum circulating levels of intercellular adhesion molecule-1 (ICAM-1) in B-cell chronic lymphocytic leukemia: Implications for prognosis. *Leuk Res* **19**: 573–580

Pignatelli M and Vessey CJ (1994) Adhesion molecules: Novel molecular tools in tumor pathology. *Hum Pathol* **25**: 849–856

Pirisi M, Falleti E, Fabris C, Soardo G, Toniutto P, Vitulli D, Pezzetta F, Bortolotti N, Gonano F and Bartoli E (1994) Circulating intercellular adhesion molecule-1 (cICAM-1) concentration in liver disease: Relationship with cholestasis and functioning hepatic mass. *Am J Clin Pathol* **102**: 600–604

Ponta H, Sleeman J and Herrlich P (1994) Tumour metastasis formation: cell-surface proteins confer metastasis-promoting or -suppressing properties. *Biochim Biophys Acta* **1198**: 1–10

Shimizu Y, Minemura M, Tsukishiro T, Kashii Y, Miyamoto M, Nishimori H, Higuchi K and Watanabe A (1995) Serum concentration of intercellular adhesion molecule-1 in patients with hepatocellular carcinoma is a marker of the disease progression and prognosis. *Hepatol* **22**: 525–531

Shino Y, Nakatani K, Watanabe A, Okumura T, Yamada Y, Yano T and Nakano H (1991) Expression of E-cadherin in human gastric carcinomas. *Gastroenterol Jpn* **26**: 231

Shino Y, Watanabe A, Yamada Y, Tanase M, Yamada T, Matsuda M, Yamashita J, Tatsumi M, Miwa T and Nakano H (1995) Clinicopathologic evaluation of immunohistochemical E-cadherin expression in human gastric carcinomas. *Cancer* **76**: 2193–2201

Smith MR, Biggar S and Hussain M (1995) Prostate-specific antigen messenger RNA is expressed in non-prostate cells: Implications for detection of micrometastases. *Cancer Res* **55**: 2640–2644

Srivastava MD, Srivastava A and Srivastava BI (1994) Soluble interleukin-2 receptor, soluble CD8 and soluble intercellular adhesion molecule-1 levels in hematologic malignancies. *Leuk Lymphoma* **12**: 241–251

Staunton DE, Dustin ML, Erickson HP and Springer TA (1990) The arrangement of the immunoglobulin-like domains of ICAM-1 and the binding sites for LFA-1 and rhinovirus. *Cell* **61**: 243–254

Suzuki Y, Ohtani H, Mizoi T, Takeha S, Shiiba K, Matsuno S and Nagura H (1995) Cell adhesion molecule expression by vascular endothelial cells as an immune/inflammatory reaction in human colon carcinoma. *Jpn J Cancer Res* **86**: 585–593

Takeichi M (1993) Cadherins in cancer: Implications for invasion and metastasis. *Curr Opin Cell Biol* **5**: 806–811

Thomson AW, Satoh S, Nüssler AK, Tamura K, Woo J, Gavaler J and Van Thiel DH (1994) Circulating intercellular adhesion molecule-1 (ICAM-1) in autoimmune liver disease and evidence for the production of ICAM-1 by cytokine-stimulated human hepatocytes. *Clin Exp Immunol* **95**: 83–90

Torii A, Harada A, Nakao A, Nonami T, Ito M and Takagi H (1993) Expression of intercellular adhesion molecule-1 in hepatocellular carcinoma. *J Surg Oncol* **53**: 239–242

Tsujisaki M, Imai K, Hirata H, Hanzawa Y, Masuya J, Nakano T, Sugiyama T, Matsui M, Hinoda Y and Yachi A (1991) Detection of circulating intercellular adhesion molecule-1 antigen in malignant diseases. *Clin Exp Immunol* **85**: 3–8

Viac J, Gueniche A, Faure M and Claudy A (1993) Soluble intercellular adhesion molecule 1 (sICAM-1) and malignant melanoma. *Cancer Lett* **72**: 191–194

Wittig BM, Kaulen H, Thees R, Schmitt C, Knolle P, Stock J, Meyer ZUM Buschenfelde KH and Dippold W (1996) Elevated serum E-selectin in patients with liver metastases of colorectal cancer. *Eur J Cancer* **32A**: 1215–1218

Wu CW, Wang SR, Chao MF, Wu TC, Lui WY, P'eng FK and Chi CW (1996) Serum interleukin-6 levels reflect disease status of gastric cancer. *Am J Gastroenterol* **91**: 1417–1422

Zetter BR (1993) Adhesion molecules in tumor metastasis. *Semin Cancer Biol* **4**: 219–229

Zhou H, Fuks A, Alcaraz G, Bolling TJ and Stanners CP (1993*a*) Homophilic adhesion between Ig superfamily carcinoembryonic antigen molecules involves double reciprocal bonds. *J Cell Biol* **122**: 951–960

Zhou H, Stanners CP and Fuks A (1993*b*) Specificity of anti-carcinoembryonic antigen monoclonal antibodies and their effects on CEA-mediated adhesion. *Cancer Res* **53**: 3817–3822

© Cancer Research Campaign 1997